# Progressive Decrease in the Potential Usefulness of Meningococcal Serogroup B Vaccine (4CMenB, Bexsero®) in Gipuzkoa, Northern Spain

**DOI:** 10.1371/journal.pone.0116024

**Published:** 2014-12-26

**Authors:** Emilio Pérez-Trallero, Olatz Esnal, José M. Marimón

**Affiliations:** 1 Microbiology Service and Reference Laboratory for Meningococcal Infections of the Basque Country, Hospital Donostia-IIS Biodonostia, San Sebastián, Spain; 2 Biomedical Research Centre Network for Respiratory Diseases (CIBERES), San Sebastián, Spain; 3 Faculty of Medicine, University of the Basque Country UPV/EHU, San Sebastián, Spain; Melbourne School of Population Health, Australia

## Abstract

The effectiveness of a vaccine is determined not only by the immunogenicity of its components, but especially by how widely it covers the disease-causing strains circulating in a given region. Because vaccine coverage varies over time, this study aimed to detect possible changes that could affect vaccine protection during a specific period in a southern European region. The 4CMenB vaccine is licensed for use in Europe, Canada, and Australia and is mainly directed against *Neisseria meningitidis* serogroup B. This vaccine contains four main immunogenic components: three recombinant proteins, FHbp, Nhba and NadA, and an outer membrane vesicle [PorA P1.4]. The allelic distribution of FHbp, Nhba, NadA, and PorA antigens in 82 invasive isolates (B and non-B serogroups) isolated from January 2008 to December 2013 were analyzed. 4CMenB was likely protective against 61.8% and 50% of serogroup B and non-B meningococci, respectively, in the entire period, but between 2012 and 2013, the predicted protection fell below 45% (42.1% for serogroup B isolates).The observed decreasing trend in the predicted protection during the 6 years of the study (*Χ* ^2^ for trend  = 4.68, *p* = 0.03) coincided with a progressive decrease of several clonal complexes (e.g., cc11, cc32 and cc41/44), which had one or more antigens against which the vaccine would offer protection.

## Introduction

*Neisseria meningitidis* is the first cause of bacterial meningitis and a frequent cause of severe sepsis worldwide. Of the 12 meningococcal serogroups described so far, six are mostly related to human invasive disease (A, B, C, X, W-135 and Y), serogroup B being the most common in developed countries [1].

Nowadays, there are effective polysaccharide conjugate vaccines against serogroups A, C, W-135 and Y, both as monovalent and as tetravalent vaccines (ACWY) but there is no conjugate vaccine against serogroup B. This is due to the similarity of the polysaccharide structure of serogroup B with the polysialic acid present in human glycoproteins, this similarity making the polysaccharide poorly immunogenic. However, for *N. meningitidis* serogroup B, there are vaccines that specifically target porin A (PorA), one of the major outer-membrane proteins. Although these vaccines have been effective in outbreaks [2], [3], they are not universal as they only target specific PorA types [4].

New technologies such as reverse vaccinology [5], [6] have facilitated the discovery of novel *N. meningitidis* antigens with high antigenic value, including factor H binding protein (FHbp), heparin binding Neisserial antigen (Nhba), and Neisserial adhesin A (NadA). The Bexsero (4CMenB) vaccine is licensed for use in Europe, Canada, and Australia and contains four main immunogenic components: three recombinant proteins FHbp, Nhba, and NadA and an outer membrane vesicle [PorA P1.4]. Although mainly directed at serogroup B meningococci, this vaccine can also protect against infections caused by isolates of other serogroups expressing one or more of the antigens included in the vaccine [7], [8]. The peptide variants included in this vaccine are B1.1 for FHbp, subvariant 2 for Nhba, variant 3 for NadA, and P1.4 for PorA. The B1.1 component of FHbp is highly immunologically cross-reactive with other B1 subvariants [9].

The effectiveness of a vaccine is not only determined by the immunogenicity of its components, but especially by how widely it covers disease-causing strains circulating in a particular region. Therefore, the aim of this study was to analyze the allelic distribution of FHbp, Nhba, NadA, and PorA antigens in invasive isolates (of serogroup B and of other serogroups) from a region of southern Europe in order to assess the potential coverage of the Bexsero vaccine against meningococcal disease within this region.

## Material and Methods

All isolates causing invasive meningococcal disease in the province of Gipuzkoa from January 2008 to December 2013 were included in the study. Only one isolate per patient was included. Gipuzkoa has about 700,000 inhabitants and is located in the north of Spain (Basque Country), bordered by the Bay of Biscay and France to the north.

The capsular serogroup was identified by latex agglutination with group-specific antibodies against capsular polysaccharides of serogroups A, B, C, W135, X, Y and Z (Murex Biotech Ltd., Dartford, England). Non-serogroupable isolates were genogrouped using a specific real-time polymerase chain reaction (PCR) using primers specific for the *siaD* and *orfA* genes [10]. Multilocus sequence typing (MLST) and identification of clonal complexes (cc) were carried out according to the *N. meningitidis MLST* database (http://pubmlst.org/neisseria/).

The allelic distribution of vaccine antigens in the meningococcal isolates was deduced from the translated sequence of their encoding genes. In this work we assumed that if 4CMenB genes encoding for vaccine antigens (GEVA) were detected in an isolate, they always expressed the antigenic proteins.

*fHbp*, *nhba*, and *nadA* typing was performed as previously proposed and described [11], [12]. The sequencing and subsequent comparison of the deduced amino acid sequences for *fHbp*, *nhba*, *nadA*, and *porA* was carried out according to the above-mentioned *N. meningitidis* MLST database. The PorA-type was determined through amplification of two fragments of the *porA* coding for the VR1 and VR2 regions [13] and subsequent comparison of the deduced amino acid sequence with PorA types available at the NM PorA variable region database (http://pubmlst.org/neisseria/).

The *Χ* ^2^ test for trends was used to compare the distribution of isolates with vaccine antigens during the 6 years of the study (2008–2013).

In the present study, no human experimentation was conducted, with all studies carried out on microorganisms obtained from samples that had previously been sent to our laboratory for routine diagnosis. The data discussed in this study were associated with *N. meningitidis* strains, and no patient information was used other than age, gender, and outcome. Conjugated vaccine against serogroup C meningococci was included in Gipuzkoa in 2000 for children at 2, 4 and 6 months of age. In 2005, the vaccine was also introduced for people between 11 and 20 years old, reaching coverage of 84.6% in this population group. Publication of these results was approved by the Ethics Committee for Clinical Research of the Gipuzkoa health region.

## Results

Between January 2008 and December 2013, meningococcal isolates from 82 patients with invasive disease were available for phenotypic and genotypic characterization. Total confirmed cases in Gipuzkoa included 91 episodes, 9.9% of them diagnosed only by PCR without culture isolation (3 serogroup B, 2 serogroup C, 1 serogroup W-135 and 3 non serogrouped). Most cases were observed in the cool months, 68.3% between November and May and especially between January and March, that comprised 50% of the cases. As in most series, the higher annual incidence rates were observed in children younger than 5 years of age (mean 18.2 cases per 100,000, range 11–27.6).

**Figure 1:**
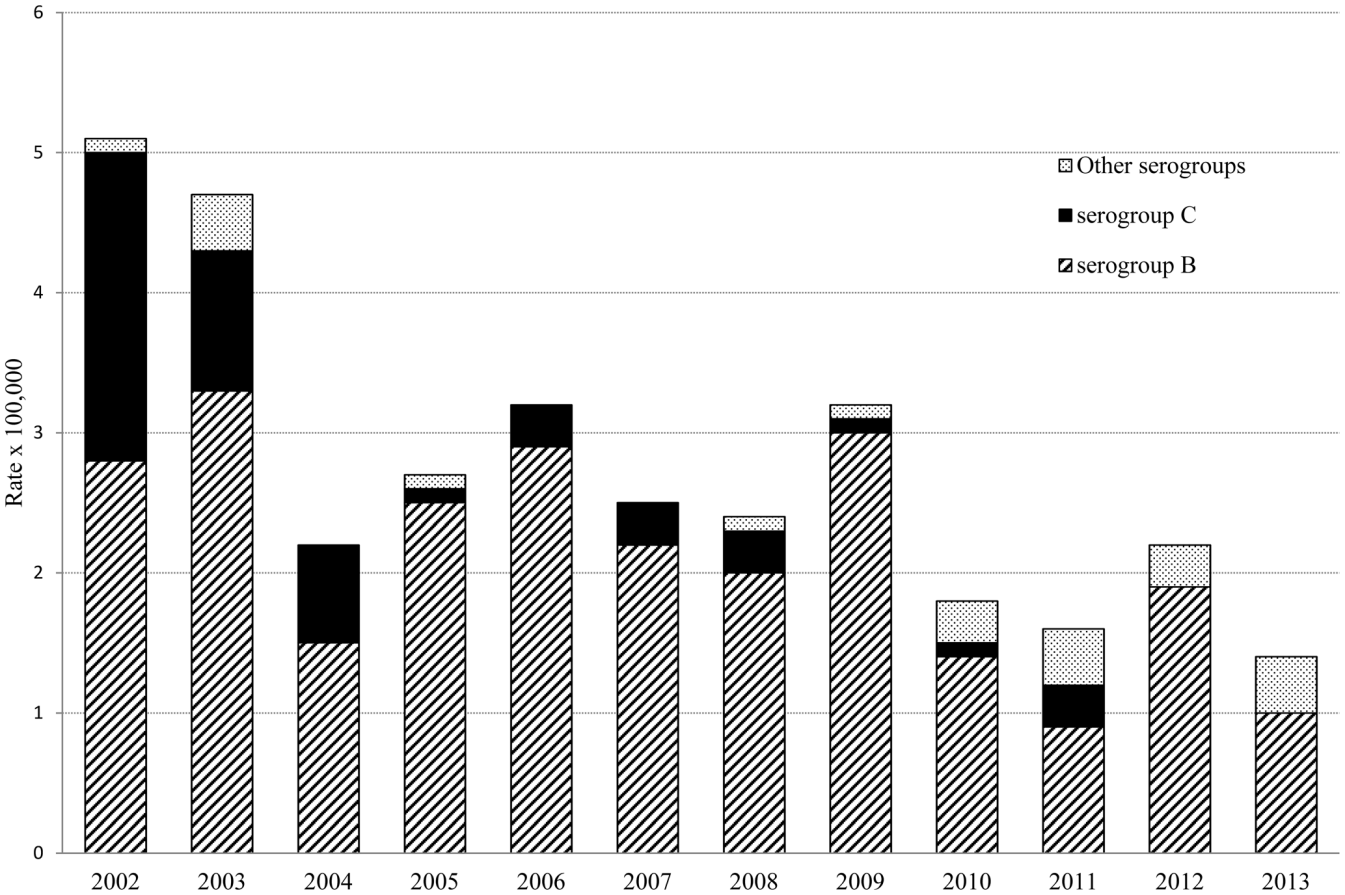
Incidence rates of meningococcal disease and serogroups distribution in Gipuzkoa, northern Spain, during a 12 years period (2002–2013).

In the past two decades, Gipuzkoa has changed from being a high to moderate endemic region (rates >5×100,000 inhabitants in the early 2000s) to a moderate to low endemic region (rates ≤2×100,000 inhabitants from 2010 on) (Fig. 1). Only 6 cases of meningococcal serogroup C disease were detected in the 6 years study period (2008–2013) as compared with 64 cases occurred in the 6 years previous to the introduction of meningococcal serogroup C conjugate vaccine (1994–1999). From 2008 to 2013, annual distribution of cases corresponded to a low to moderate endemic region, without outbreaks or clusters worthy of mention.

Forty-one isolates were collected from blood-cultures, 33 from cerebrospinal fluid (CSF), and the remaining eight isolates were isolated both in the blood and CSF of the same patient.

Of the 82 patients, 45 (55%) were female and 37 (45%) male. More than half of the isolates were collected from patients younger than 10 years old (*N* = 43, 52%), while the second age group with the highest number of isolates consisted of people aged ≥65 years old (*N* = 13, 15.8%). Six patients (7.3%) died from meningococcal infection, all of them with sepsis: 1 children aged 4 years without previous disease, 2 teenagers 15 and 16 years old (both immunosuppressed), one young adult 27 years old (paroxysmal nocturnal hemoglobinuria) and two elderly patients aged 83 years old.

Most of the 82 isolates belonged to serogroup B (*N* = 68, 83%), although serogroups C (*N* = 4, 5%), Y (*N* = 6, 7%), X (*N* = 2, 2.5%), and W-135 (*N* = 2, 2.5%) were also present.

### Analysis of the genes encoding the antigens included in the 4CMenB vaccine (GEVA)

*fHbp* could be characterized in all 82 isolates included in the study, with 50% (*N* = 41) belonging to sub-family/variant B1, 30.5% (*N* = 25) to sub-family/variant A2 and 19.5% (*N* = 16) to sub-family/variant A3. The analysis of GEVA demonstrated that 41 isolates showed potential vaccine coverage for the FHbp antigen (41 variant B1 isolates, 50%), of which 36 were serogroup B (36/68, 52.9%). Only three of the 41 *fHbp* variant B1 isolates showed subvariant B1.1.

Among the 36 serogroup B isolates with the *fHbp* variant B1, 21 were collected in 2008–2009, nine in 2010–2011, and six in 2012–2013 (Table 1).

**Table 1:** Genes that produce the antigens included in the 4CMenB vaccine detected among invasive *Neisseria meningitidis* isolates in Gipuzkoa, northern Spain (2008–2013).

Year	2008		2009		2010		2011		2012		2013		2008–2013	
(number invasive isolates)	(*N* = 15)		(*N* = 21)		(*N* = 13)		(*N* = 10)		(*N* = 15)		(*N* = 8)		(*N* = 82)	
Serogroups	B	others	B	others	B	others	B	others	B	others	B	others	B	others
	*N* = 14	*N* = 1	*N* = 19	*N* = 2	*N* = 10	*N* = 3	*N* = 6	*N* = 4	*N* = 13	*N* = 2	*N* = 6	*N* = 2	*N* = 68	*N* = 14
***fHbp*** ** B1**	7	1 (C)	13	1 (C)	5	1 (C)	3	1 (C)	4	0	2	1 (Y)	34	5
***fHbp*** ** B1** ***+ nhba2***	0	0	0	0	1	0	0	0	0	0	0	0	1	0
***fHbp*** ** B1** ***+ porA*** ** VR2 4**	1	0	0	0	0	0	0	0	0	0	0	0	1	0
***nhba2***	0	0	0	0	1	0	1	0	0	0	1		3	0
***nadA 1,2/3***	0	0	0	1 (Y)	0	0	0	0	1	0	0	0	1	1
***porA*** ** VR2 4**	2	0	0	0	0	0	0	0	0	1 (W)	0	0	2	1
**Total potential covered by 4CMenB vaccine**	10	1	13	2	7	1	4	1	5	1	3	1	42	7

*nhba* was also present in all 82 isolates, but only four of them (all serogroup B isolates) had Nhba subvariant 2, the antigen included in the 4CMenB vaccine, for which the potential coverage was 4.9% (4/82) for all meningococcal invasive isolates and 5.9% (4/68) for serogroup B isolates.

Amplification of *nadA* failed in 71% (58/82) of isolates. Of the 24 isolates with this antigen, only two isolates from 2009 and 2012 had variant 3 (included in the vaccine) or variants 1 and 2 (which cross-react with variant 3).The potential coverage of the NadA antigen was 2.4% (2/82) for all meningococcal invasive isolates and 1.5% (1/68) for serogroup B isolates.

*porA* was detected in all *N. meningitidis* isolates, with only four isolates (three serogroup B) coding for the VR2 4 region, which determines the P1.4 antigen included in the vaccine. One isolate with the *porA* coding for the VR2 4-1 region was not included because this antigen does not react with P1.4 monoclonal antibody [14]. The potential coverage of the 4CMenB vaccine for the P1.4 antigen was 4.9% for all serogroups and was 4.4% for serogroup B invasive isolates.

Two isolates had two GEVA: one had the *fHbp* variant B1 plus *porA* VR2 4 and the other the *fHbp* variant B1 plus *nhba2* genes (Table 1).

Overall, the potential coverage of the 4CMenB vaccine was 59.8% for all meningococcal invasive isolates but progressively decreased during the study period. Coverage significantly decreased from 72.2% in 2008–2009, to 56.5% in 2010–2011, and to 43.5% in 2012–2013, revealing an annual decreasing trend during the 6 years of the study (*Χ* ^2^ for trend  = 4.68, *p* = 0.03).

When only serogroup B isolates were analyzed, 69.7% were theoretically covered by the vaccine in 2008–2009, but this percentage decreased to 42.1% in 2012–2013 (*p* = 0.051). This decreased coverage coincided with a progressive decline in our region of several cc with GEVA (especially cc11 and cc41/44).

### Relationship between vaccine antigens and clonal complexes

Genotyping showed the presence of 15 different cc described in the *N. meningitidis* MLST data base (http://neisseria.org/nm/typing/mlst/). Five isolates could not be included in any cc (ST6421, ST7306 and another 3 new STs) and one isolate (serogroup B, ST4954) was included in a new e-Burst group defined by us [4] but without formal cc inclusion to date. The most frequent cc were 213 (*N* = 14), 269 (*N* = 12), 11 (*N* = 10), and 60 (N = 9) (Table 2). The distribution of the cc between 2008 and 2013 was uneven both in time and age groups and no cc was related to higher mortality. Only cc269 was present throughout the study period (Table 2). The largest cluster of cases caused by a single clone occurred in 2009, with five ST11 meningococci: 4 serogroup B (in 2 young adults, 1 child of six years and an elderly patient) and one serogroup C (in a 9 years old child) with no apparent epidemiological relation. Also in 2009, six cc213 isolates were detected, but only three of them had the same ST (3 ST3496, and 1 each ST4224, ST8756 and ST10121).

**Table 2:** Number and percentage of invasive isolates with genes encoding proteins targeted by the Bexsero vaccine according to the isolated clonal complexes (CC). Gipuzkoa, (Basque Country, northern Spain).

	years							
Clonal complexes	2008–2009		2010–2011		2012–2013		Total 2008–2013	
**cc213**	5/8	62.5%	0/1	0%	1/5	20%	6/14	42.9%
**cc269**	1/4	25%	1/4	25%	0/4	0%	2/12	16.7%
**cc11**	7/7	100%	2/3	66.7%	-	-	9/10	90%
**cc60**	2/2	100%	4/4	100%	3/3	100%	9/9	100%
**cc32/ET-5**	3/3	100%	1/1	100%	2/2	100%	6/6	100%
**cc41/44**	2/2	100%	3/3	100%	1/1	50%	6/6	100%
**cc461**	1/2	50%	-	-	0/2	0%	1/4	25%
**Other cc** a **or singletons**	5/8	62.5%	2/7	28.6%	3/6	50%	10/21	47.6%
**Total isolates**	26/36	72.2%	13/23	56.5%	10/23	43.5%	49/82	59.8%

a cc103 (n = 3); cc35 (*N* = 2); cc162 (*N* = 2); cc167 (*N* = 2); cc174 (*N* = 2); cc750 (*N* = 2); cc22 (*N* = 1); cc1117 (*N* = 1); singletons (*N* = 6).

The presence of GEVA among isolates of a specific cc was diverse (Table 2). In some cc (cc11, cc60, cc32, cc41/44, cc103, cc162, and cc1117), ≥90% of isolates were theoretically covered by the 4CMenB vaccine. No isolates of cc167, cc35, cc750 or cc22 showed GEVA.

## Discussion

Recent publications have reported the potential coverage of the 4CMenB vaccine for Spain and other European countries [9], [15]–[17]. Because the coverage in a specific region can vary over time due to distinct clonal circulation, this study aimed to detect possible changes that could affect vaccine protection during a specific period. Studies evaluating this protective effect have been performed using the MATS (Meningococcal Antigen Typing System) ELISA provided by Novartis or, as in the present study, by analyzing the allelic distribution of the four main antigens included in the 4CMenB vaccine. We assumed that the presence of *fHbp* B1, *nhba*2, *nadA 1,2/3* and *porA* VR2 4, once expressed, is a prerequisite for the protective immune response induced by vaccination. Although the immune response to the 4CMenB vaccine is not limited to the immunogenicity of its four major antigens, these are the main inductors of protection.

The FHbp and Nhba antigens are present in virtually all *N. meningitidis*, although their expression level may vary among isolates [18]–[20] and not all their variants were included in the vaccine. In the present study, *fHbp* and *nhba* were detected in all 82 invasive isolates, but the variant predicted to confer protection (FHbp B1 and Nhba 2) was detected in only half and less than 5% of isolates, respectively. Some authors suggested that Nhba variants 3, 20, 21 and 113 could also confer protection, due to cross-reactivity with Nhba variant 2 (16,17). Taking into account this latter criterion, only one more isolate, having Nhba variant 21 and isolated in 2012, would have been included. *nadA* was absent in most isolates, a fairly common occurrence, especially in some CCs [21] and, in the present study, only two isolates had the vaccine antigenic variants 1, 2, and 3. Finally, only four isolates codified the PorA VR2 4 region that produces the P1.4 antigen included in the vaccine.

Given that the presence of each of the four antigens would stimulate the immune response of vaccinated patients, almost 60% of patients could have been protected by 4cMenB vaccination (61% including the Nhba variant 21). However, the theoretical protection between 2012 and 2013 fell below 45% (48% including the Nhba variant 21).

Percentages of theoretical protection greater than 75% have been reported for Europe as a whole and for some other European countries [9], [15]–[17]. A different theoretical coverage could have been obtained, depending on the study period. In a European study [17], lower protective figures were obtained for Spain and the Czech Republic, the only two countries with isolates obtained after 2008. Our strains were not included in the study of Vogel et al (17) but showed the same distribution of serogroups, cc and theoretic 4CMenB protection figures as the Spanish meningococcal isolates collected between 2008 and 2010 of that study. *N. meningitidis* is very prone to the exchange of genetic material and it is unsurprising that the meningococcal population changes over time [22]. The reduction in 4CMenB vaccine protection could have been due to decreased circulation of clones harboring GEVA or to changes in GEVA in some isolates of the same clone. The most frequently found serogroup B meningococcal clones during the previous decade in our region [4] and in other European countries [23] were cc in which most of the isolates had GEVA (e.g., cc41/44, cc32, cc11). The decreasing trend in the potential 4CMenB vaccine protection observed during the 6 years of the present study was influenced by the decrease in the circulation of CC whose isolates possessed GEVA (cc60 complex, cc32 complex ET-5, cc11 complex and cc41/44 complex), although the fact of belonging to a certain cc is insufficient to predict antigenic profile. Nine of the 10 cc11 isolates belonged to the ST11 hypervirulent clone and all had the FHbp B1 antigen against which the vaccine would confer protection. cc269 was highly prevalent in Europe and some of its STs, such as ST269, have been reported to possess vaccine antigens in all or almost all isolates [24], [25]. However, in the present study, most cc269 isolates were ST1163 and they lacked GEVA. Over time, changes are common in both the circulation of clones and in the genetic information within a single clone.

The incidence of meningococcal disease in our region has substantially declined in the last decade, and most recent circulating isolates lacked antigens against which vaccinated people would have been protected. In the current situation, and considering the high incidence of fever from the vaccine [26], the introduction of the 4CMenB vaccine in our community does not seem to achieve the necessary priority to be recommended. If an outbreak caused by a hypervirulent clone were to occur, especially if the clone was the ST11 clone, the vaccine would be extremely useful.
